# Parthenocarpy-related genes induced by naphthalene acetic acid in oil palm interspecific O × G [*Elaeis oleifera* (Kunth) Cortés × *Elaeis guineensis* Jacq.] hybrids

**DOI:** 10.3389/fgene.2023.1099489

**Published:** 2023-03-20

**Authors:** Carmenza Montoya, Fernan Santiago Mejia-Alvarado, David Botero-Rozo, Ivan Mauricio Ayala-Diaz, Hernan Mauricio Romero

**Affiliations:** ^1^ Oil Palm Biology and Breeding Research Program, Colombian Oil Palm Research Center—Cenipalma, Bogotá, Colombia; ^2^ Department of Biology, Universidad Nacional de Colombia, Bogotá, Colombia

**Keywords:** oil palm, interspecific O × G hybrids, NAA, auxins, transcriptome, gene coexpression networks, parthenocarpy

## Abstract

Parthenocarpy is the development without fertilization of seedless fruits. In the oil palm industry, the development of parthenocarpic fruits is considered an attractive option to increase palm oil production. Previous studies have shown the application of synthetic auxins in *Elaeis guineensis*, and interspecific O×G hybrids (*Elaeis oleifera* (Kunth) Cortés × *E. guineensis* Jacq.) induces parthenocarpy. The aim of this study was to identify the molecular mechanism through transcriptomics and biology system approach to responding to how the application of NAA induces parthenocarpic fruits in oil palm O×G hybrids. The transcriptome changes were studied in three phenological stages (PS) of the inflorescences: i) PS 603, pre-anthesis III, ii) PS 607, anthesis, and iii) PS 700, fertilized female flower. Each PS was treated with NAA, Pollen, and control (any application). The expression profile was studied at three separate times: five minutes (T0), 24 hours (T1), and 48 h post-treatment (T2). The RNA sequencing (RNA seq) approach was used with 27 oil palm O×G hybrids for a total of 81 raw samples. RNA-Seq showed around 445,920 genes. Numerous differentially expressed genes (DEGs) were involved in pollination, flowering, seed development, hormone biosynthesis, and signal transduction. The expression of the most relevant transcription factors (TF) families was variable and dependent on the stage and time post-treatment. In general, NAA treatment expressed differentially more genes than Pollen. Indeed, the gene co-expression network of Pollen was built with fewer nodes than the NAA treatment. The transcriptional profiles of Auxin-responsive protein and Gibberellin-regulated genes involved in parthenocarpy phenomena agreed with those previously reported in other species. The expression of 13 DEGs was validated by RT-qPCR analysis. This detailed knowledge about the molecular mechanisms involved in parthenocarpy could be used to facilitate the future development of genome editing techniques that enable the production of parthenocarpic O×G hybrid cultivars without growth regulator application.

## Introduction

Parthenocarpy is the development of seedless fruits without fertilization. Parthenocarpy is commercially exploited because fruit seeds could be undesirable due to their hard texture, bitter taste, and toxic compounds and allergens that can affect palatability. Therefore, seedless fruits are desirable to improve the quality of fresh and processed fruits. Furthermore, replacing seeds and seed cavities with edible tissue attracts consumers (Dhatt and Kaur, 2016). Developing parthenocarpic fruits is considered an attractive option to increase palm oil production. It allows for higher oil content by eliminating the kernel and increasing the mesocarp, resulting in more oil stored in the fruit. Previous studies have shown the efficacy of auxins to induce parthenocarpy in oil palm interspecific O × G [*E. oleifera* (Kunth) Cortés × *E. guineensis* Jacq.] hybrids without interfering with fruit development, oil synthesis, fatty acid profile, oil quality, or bunch components (Daza et al., 2020). Using the synthetic auxin naphthalene acetic acid (NAA), it is possible to reach more than 10 tons per hectare per year of High-Oleic Palm Oil (HOPO) without increasing the planted oil palm area by the induction of parthenocarpic fruits in the O × G hybrids (Romero et al., 2021).

NAA is widely used in Colombia and Ecuador, where interspecific O × G hybrids are planted in large extensions to replace the African oil palm, *E. guineensis*. The use of O × G hybrids is increasing because the African oil palm is susceptible to bud rot disease (BR) (Navia et al., 2014; Avila-Diazgranados et al., 2016), the most limiting factor of oil palm production in America (Sundram and Intan-Nur, 2017). The O × G hybrids resist BR, have high productivity potential, and produce a high-quality oil rich in oleic acid (Romero et al., 2022). However, the oil extraction rate under natural pollination conditions is low due to limitations in bunch filling that may be associated with low pollen viability and germinability and indehiscent peduncular bracts in female inflorescences that make pollen entry to the flowers difficult (Hormaza et al., 2012). Under natural pollination, the O × G oil potential is much lower than the African oil palm. However, the oil potential is increased with assisted pollination, a mandatory practice in the O × G interspecific hybrids (Rincon et al., 2013). It is even higher when NAA is applied to induce parthenocarpic fruits (Romero et al., 2021).

The idea of applying growth regulators to oil palm began 50 years ago. However, a genuine interest was shown recently due to its potential applicability. In African oil palm, the exogenous application of the synthetic auxin 2,4,5-tri-chlorophenoxy propionic acid (2,4,5-TP) has been associated with a seedless phenotype. The expression of genes related to the synthesis of auxins and gibberellins like EgGH3.8 (indole -3-acetic acid (IAA)-amido synthetase GH3.8), EgGH3.1 (IAA-amido synthetase GH3.1), EgARG7 (IAA induced ARG7 like), EgTAA3 (tryptophan amino transferase-related protein 3-like) and EgFMO1 (flavin-containing monooxygenase) were associated with parthenocarpic fruit development (Somyong et al., 2018). On the other hand, Htwe et al. (2022) studied gene expression in *E. guineensis* under natural pollination conditions and boron applications. Gene expression results showed eight genes associated with programmed cell death (PCD) and three groups of genes related to PCD: 4-coumarate-CoA ligase (4CL), S-RNase, and MADS-box. These findings suggest that a synergic upregulation of 4CL and MADS-box TFs activated the expression of PCD genes in the pollen tube, leading to fertilization failure and the seedless phenotype. Although these genes exhibited differential expression between treated and untreated fruits, more research is needed to identify which genes are involved in parthenocarpy and their function in the metabolic pathways of embryo formation.

No transcriptome studies show gene expression patterns in the O × G hybrid when NAA induces parthenocarpic fruit formation. Thus, this study aims to identify genes that respond to the application of NAA, to understand the molecular and genetic basis of induced parthenocarpic fruits. In the long term, the goal is to use genetic editing methodologies that could facilitate the production of parthenocarpic O×G hybrid cultivars to produce fruits without the external application of growth regulators.

## Materials and methods

This research was carried out at the Palmar de la Vizcaína Experimental Field (CEPV) of the Colombian Oil Palm Research Centre - Cenipalma, located in the rural area of Barrancabermeja, Santander, Colombia, at 6°58ʹ863ʹʹN and 73°42ʹ186ʹʹW. With an elevation of 102 m above sea level, an average annual rainfall of 3,472 mm, an average temperature of 29°C, and relative humidity (HR%) between 72% and 77%.

### Inflorescences collection, RNA isolation, and library construction

**FIGURE 1:**
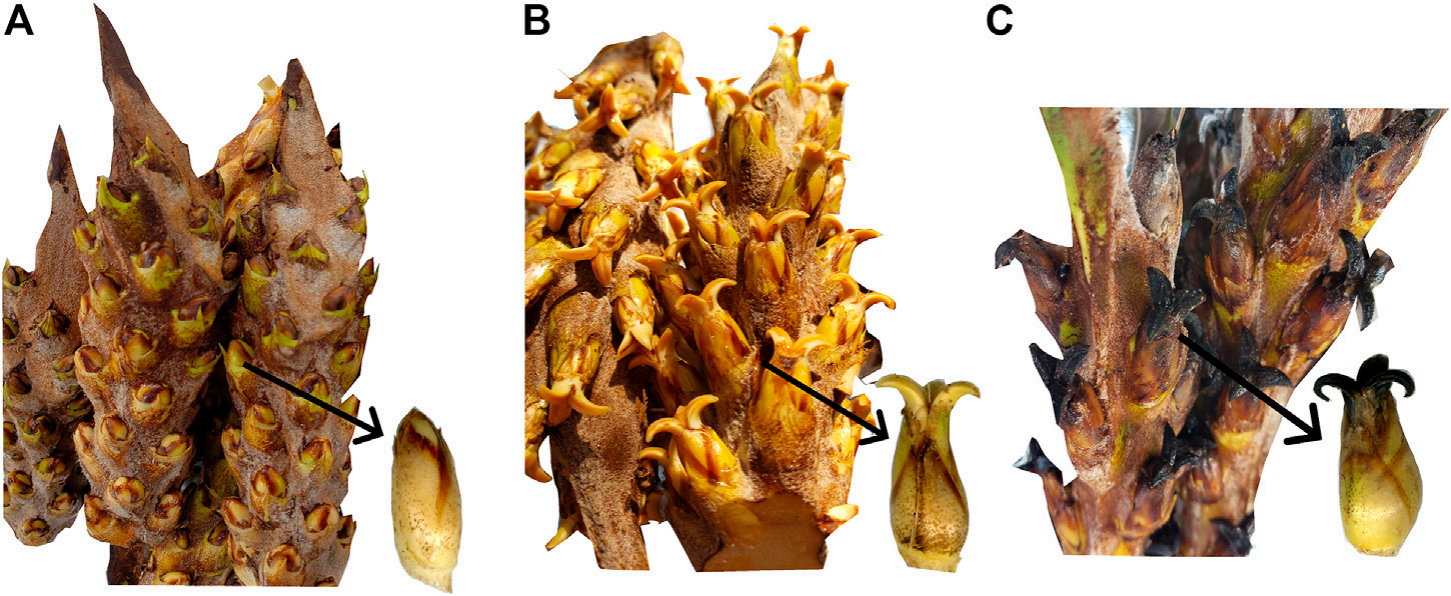
Inflorescences of oil palm interspecific O × G hybrids and single female flower by phenology states. **(A)** PS_603, **(B)** PS_607, and **(C)** PS_700.

**FIGURE 2:**
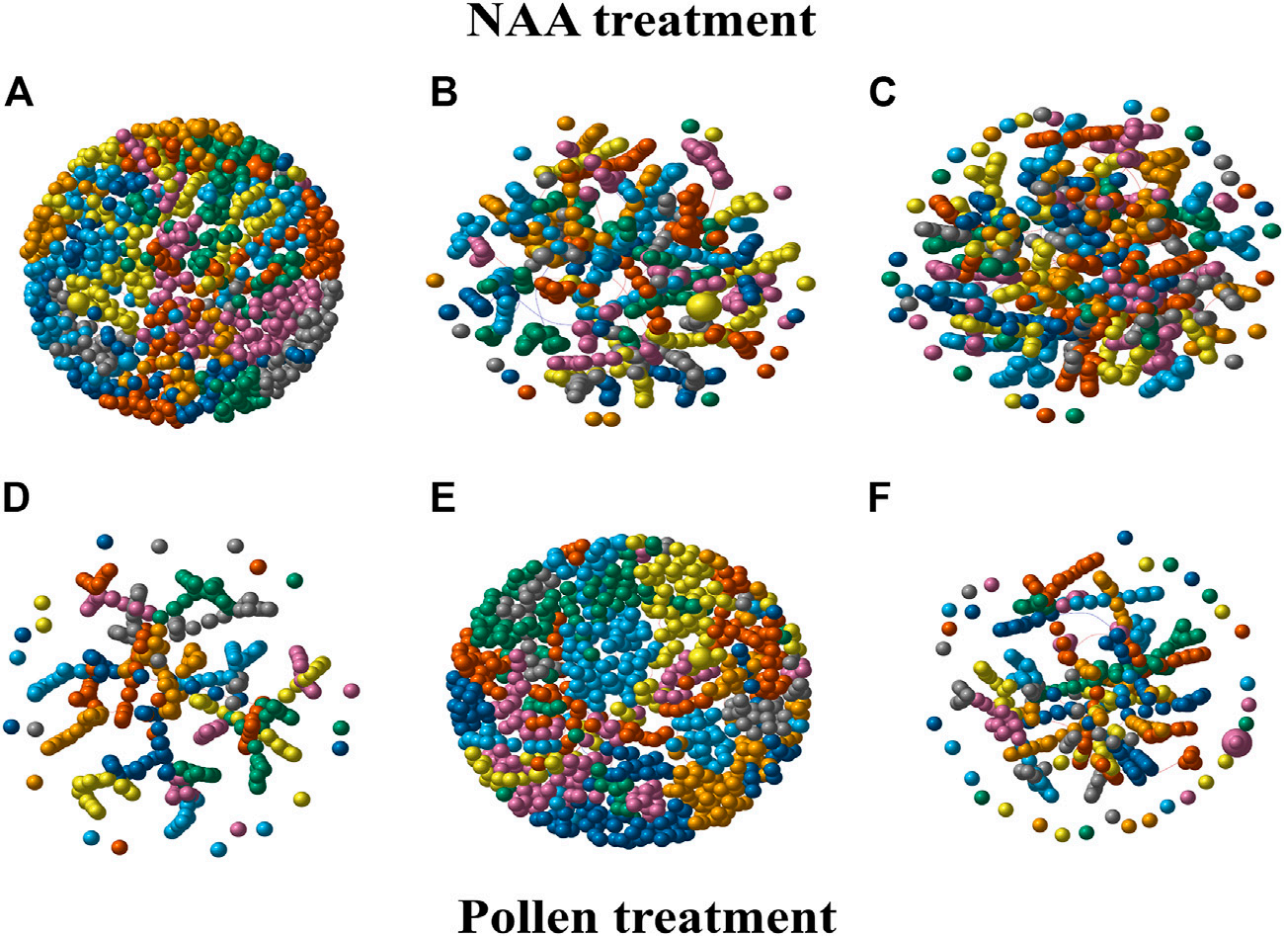
The co-expression network for each phenology stage under NAA **(A–C)** and the pollen **(D–F)** treatment. Each network was constructed with whole T0, T1, and T2 DEGs per treatment. Each node (sphere or bead-like shape) represents a gene, and groups of nodes with the same color indicate a module of genes. Black edges represent a direct correlation between genes, and red lines inverse correlation. The nodes’ size is proportional to the mean level expression of the gene represented by the node. The *igraph* R package was used to construct the phenology stage-specific co-expression networks under NAA and Pollen treatment.

**FIGURE 3:**
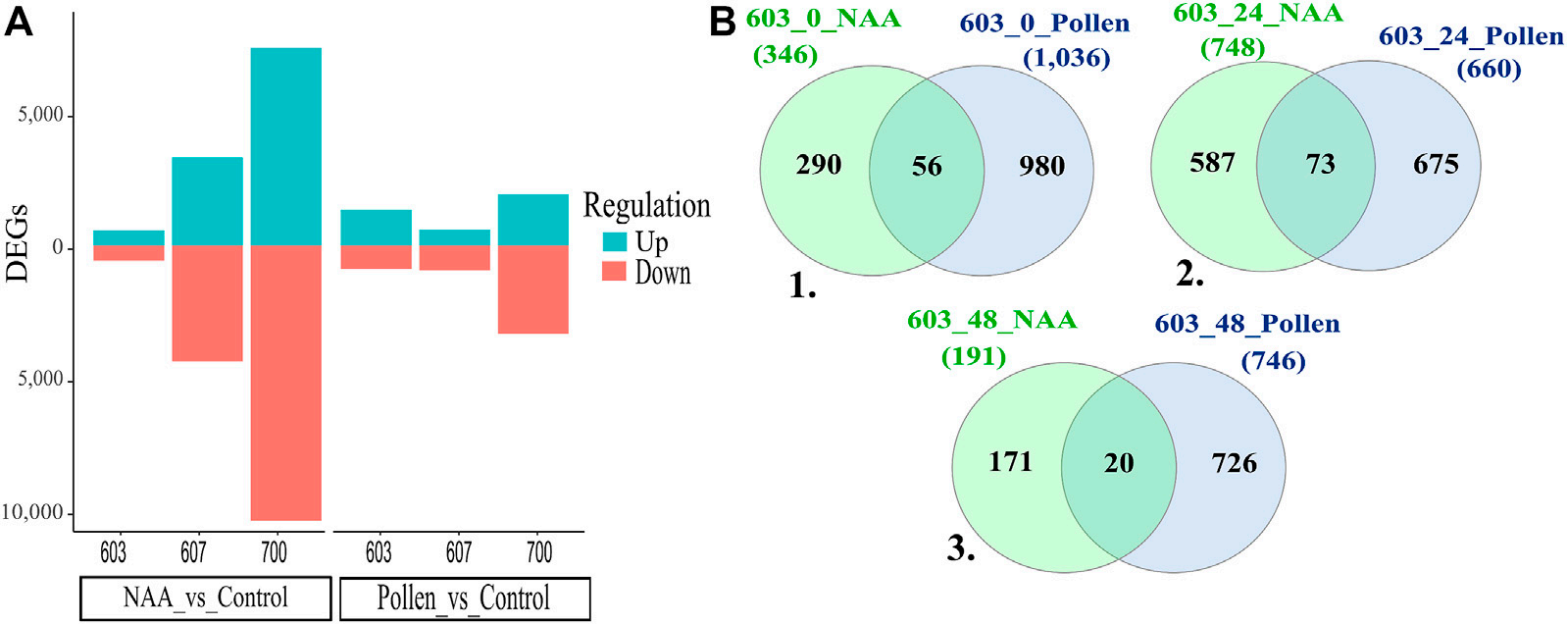
**(A)** DEGs over-expressed (Up) and under-expressed (Down). **(B)** Venn diagrams for PS 603. 1. Commons genes shared in T0 between NAA and Pollen treatment. 2. Commons genes shared in T1 between NAA and Pollen treatment. 3. Commons genes shared in T2 between NAA and Pollen treatment. Conventions: 607_48_Pollen = Phenology stage_time post treatment_treatment.

The study included 27 O × G interspecific hybrid palms (Coari × La Mé). Female inflorescences were selected in the phenological stage (PS) 601 or pre-anthesis I (Hormaza et al., 2012). Each inflorescence was enclosed in a polyester bag (PBS International, UK) to prevent cross-contamination with pollen or pollinating insects. Treatments of NAA, Pollen, and controls were applied when the isolated female inflorescences reached three different PS: 1) pre-anthesis III or PS 603 (Figure 1A), 2) anthesis or PS 607 (Figure 1B), and 3) fertilized female flower or PS 700 (Figure 1C). The phenological stages were chosen because, in PS 607 and PS 700, farmers are applying NAA solution to the O × G hybrid to obtain parthenocarpic fruits. The PS 603 was used to detect the earliest pollen and NAA signaling pathways and elucidate early differences between treatments. Three oil palms of each PS were treated with 150 mL of NAA solution (1,200 ppm), another independent three oil palms of each PS were treated with a mixture of Pollen and inert talcum powder (1:9 ratio), and finally, another three oil palms of each PS were kept without any application as control. After each treatment, samples were collected at three different times for each PS: 5 min post-treatment (T0), 24 h post-treatment (T1), and 48 h post-treatment (T2). Eighty-one samples were collected from 27 treatments and three replicates per treatment. From each inflorescence, 20 spikes were collected and immediately immersed in liquid nitrogen. The samples were homogenized with liquid nitrogen before RNA isolation. Total RNA was extracted from 100 mg of inflorescence tissue using the NucleoSpin RNA Plant and Fungi Mini kit for RNA from plants and fungi (Macherey-Nagel SAS, Dueren, Germany) according to the manufacturer’s protocol. The concentration and quality of total RNA were examined using an Agilent 2100 Bioanalyzer Instrument (Santa Clara, CA, United States). RNA samples with RIN ≥7 were precipitated with 3M sodium acetate and ethanol and sent to RNA-Seq library construction by Genohub Inc. (Austin, TX, United States). The libraries were constructed using the KAPA mRNA HyperPrep kit (Roche Diagnostics Corporation, Indianapolis, IN, United States) and sequenced in the Illumina NovaSeq 6000 platform (Illumina, Inc. San Diego, CA, United States).

### Quality control and DEGs analyses

The quality of the 81 raw samples was explored by FastQC (Andrews, 2010) and MultiQC (Ewels et al., 2016). The ten first bases were trimmed, and reads shorter than 25 were discarded with Trimmomatic (Bolger et al., 2014). Transcriptome assembly and downstream analyses were performed using the Trinity protocol, according to Haas et al. (2013). The clean reads were assembled with Trinity using *in silico* normalization by sample and default parameters (K25, maxC200, minC1, maxCV10000) (Grabherr et al., 2011). Longest open reading frames (LongORF) were predicted with TransDecoder. The transcriptome was functionally annotated through Trinotate with BLAST 2.11.0+ against the Uniprot database (Consortium, 2020); protein domains were searched with HMMER 3.3.2 against the Pfam database (Eddy, 2010; Punta et al., 2012). To evaluate the quality of the transcriptome, clean reads were mapped using Burrows-Wheeler Alignment (BWA) tool mem (Li and Durbin, 2009). Based on the lengths of the assembled transcriptome contigs, N50 length statistics were calculated.

The abundance of genes and transcripts per sample was calculated using the free-alignment method, Kallisto (Bray et al., 2016). These estimations were used for the matrix counts construction and values normalization. DEGs were statistically assessed by DESeq2 (Love et al., 2014) using the specific contrasts of treatments, PS, and time (i.e., NAA_603_T0 vs. Control_603_T0). Gene ontology enrichment was performed with Trinotate and GOseq (Young et al., 2010). Finally, an annotation report was generated with Trinotate for downstream analysis.

### Gene co-expression network reconstruction

Using the DEGs, co-expression networks were constructed for NAA and Pollen contrasts for each PS 603, 607, and 700. A *p*-value ≤0.1 and a Log2FoldChange ≥ |2| were set in the DESeq2 algorithm as thresholds for assessing significant genes. The igraph R package was used to construct the co-expression networks. Each network was built based on correlating all gene pair distances among normalized read counts (variance stabilization in DESeq2). Only sample columns of the corresponding phenomena were used (i.e., for the NAA_603 network, only columns of T0, T1, and T2 of NAA and control at PS 603 were used). The edge among genes was considered when the correlation was ≥0.8. The “edge betweenness” function identified communities or modules of genes correlated among them (Blondel et al., 2008). HUB’s genes were determined according to a HUB score using the algorithm developed by Kleinberg (1999). Additional metrics of the networks were estimated as density, diameter (Wasserman and Faust, 1994), weighted diameter, centralization degree, centralization closeness, centralization betweenness (Brandes et al., 2016), and average path length (West, 2001).

### Transcriptome and gene co-expression network analysis

To study the DEGs involved in NAA-induced parthenocarpy compared to the pollination mechanism, Venn diagrams were constructed using the web-based tool InteractiVenn (Heberle et al., 2015) to identify common and unique genes among PS (603, 607, and 700), treatment (NAA and Pollen) and time post-treatment (T0, T1, and T2). Furthermore, topological centrality measurements, such as HUB score, degree, betweenness, and closeness, were analyzed to identify HUB genes and representative modules in the co-expression network. Finally, the GO terms and categories with *p*-value ≤0.05 were significantly enriched. The overrepresented and enriched DEGs were analyzed in the REVIGO web application tool (Supek et al., 2011) to visualize how DEGs were clustered in categories of GO terms and their significance.

### Validation of DEGs by quantitative real-time PCR

To confirm RNA-Seq transcriptome, 13 genes were selected according to the differential expression among PS, highly HUB scored, and literature reports (target and reference genes sequences are listed in Supplementary Table S1). Primer sequences were designed with the program Primer3web V4.1.0 (https://primer3.ut.ee/). The RT-qPCR reaction was conducted in a 10 µL reaction of QuantiNova SYBR Green One-Step RT-qPCR kit (QIAGEN, Germany) according to the manufacturer’s instruction. Three µL of RNA (50 ng/μL) were added to each reaction as a template. The RT-qPCR was run in a ROCHE LightCycler^®^ 480 Real Time PCR System (Roche Diagnostics International AG, Rotkreuz, Switzerland). The PCR conditions were set as follows: 10 min at 50°C for reverse transcription, followed by the initial denaturation at 95°C for 2 min, then 40 cycles of denaturation at 95°C for 10 s, annealing at 58°C for 30 s and extension at 72°C for 30 s. For the melting curve analysis, the ramp rate was set at 0.06°C/s from 60°C to 95°C. Relative expression for each gene was calculated using the delta-delta of Ct method (∆∆Ct), and CYP2 and GRAS genes were utilized as normalizers. The association between RNA-Seq and RT-qPCR results was established by a correlation coefficient in R and plotted with the ggplot package.

## Results

### Transcriptome sequencing (RNA-seq) analysis

On average, the 81 raw samples had 48 million reads per sample and 7.8 billion total reads. After cleaning with Trimmomatic, around 34 million reads per sample and a total of 5.5 billion reads were obtained. 445,920 genes and 797,051 transcripts (isoforms) were assembled with Trinity. Most of the paired reads mapped correctly with BWA tool mem. At least half of the assembled bases (N50) were found in contigs at least 1,765 bases in length, 1,206, when the longest isoforms per gene were used. Sequencing generated 150 bp paired-end reads.

### Gene co-expression networks

Network metrics comparing NAA and Pollen at the three stages are summarized in Supplementary Table S2. The Pollen had a smaller number of nodes than NAA. Also, in Pollen, there was a decrease in the number of nodes from PS 603 to 607 and an increase in PS 700. In contrast, NAA networks predicted fewer nodes in PS 603 but increased around three times in PS 607 and 700; also, there was a considerable difference between these last stages (488 nodes). Accordingly, densities in the PS 607 and 700 NAA networks were smaller than in the other networks by one order of magnitude; thus, the proportion of present edges from all possible edges in the network was smaller than in different networks. The centralization closeness of NAA_603 was one order of magnitude greater than the other networks, so the inverse of the node’s average geodesic distance to other nodes in the network was the greatest in this network. This was expected since if the number of nodes is smaller, the geodesic distance among nodes is smaller; however, it is interesting to note that the Pollen_607 network had a similar number of nodes network, but it did not have a centralization closeness value in the same magnitude.

The general co-expression network analysis of NAA phenomena simultaneously activated several pathways and genes in PS 603, PS 607, and PS 700 through a modulated response (Figure 2). In the co-expression network for PS 603_NAA (Figure 2A), the under-expressed genes TRINITY_DN47848_c0_g3 and TRINITY_DN12574_c3_g1 were predicted as HUB genes. The co-expression network in PS 607_NAA (Figure 2B) was constructed from 3,145 DEGs, and the gene TRINITY_DN22399_c0_g1 (Doubtful hypothetical protein ∼ unknown_gene) was predicted as HUB gene. This gene was highly over-expressed in T0, T1, and T2. TRINITY_DN56636_c0_g2 (possible protein SCAR2-like) was found over-expressed and predicted as the HUB gene in the co-expression network for PS 700_ NAA (Figure 2C).

The co-expression network to predict the pollination mechanism (Pollen treatment) was constructed with fewer DEGs than the NAA network. It displayed less modularity and diameter compared to the NAA treatment. The PS 603 Pollen co-expression network (Figure 2D) predicted the genes TRINITY_DN63896_c2_g1 and TRINITY_DN24805_c2_g1 as HUB genes. In the PS 607_Pollen gene co-expression network (Figure 2E), TRINITY_DN91842_c1_g1 was predicted as the most critical gene in this model. Concerning the PS 700_Pollen gene co-expression network (Figure 2F), TRINITY_DN99294_c0_g1 and TRINITY_DN97297_c0_g1 (Probable metal-nicotianamine transporter YSL6) were predicted as HUBs.

### DEGs and gene ontology analysis

A comparative analysis was performed among Pollen and NAA samples in different combinations of time and phenology stages to identify DEGs. In general, NAA treatment expressed differentially more genes than Pollen (Figure 3A). PS 607 and PS 700 under NAA treatment showed a higher level of DEGs than the Pollen treatment; additionally, PS 700 presented a significant quantity of under-expressed genes.

The general comparison of PS 603 (603_T0, 603_T1, and 603_T2 of Pollen and NAA treatments) did not share common genes among times and treatments (Supplementary Figure S1). However, expression changes were observed when time and treatments were compared (i.e., NAA _603_T0 vs. Pollen _603_T0). For the PS 603_T0 comparison (Figure 3B1), 56 genes were shared, with six genes drastically changing their expression at 5 minutes (T0) post-NAA application compared to Pollen. The most relevant genes were TRINITY_DN57151_c0_g2, TRINITY_DN51871_c1_g1 (Probable receptor-like protein kinase At1g33260), and TRINITY_ DN35796_c1_g1 (Calcium uniporter protein 5, mitochondrial). Then, in the PS 603_T1 (Figure 3B2), 73 genes were shared between NAA and Pollen treatments. Here, ten genes presented changes in expression level between treatments: the most relevant genes were TRINITY_DN57151_c0_g2, TRINITY_DN57151_c0_g2 (Subtilisin-like protease SBT1.5), and TRINITY_DN46085_c0_g1. Finally, in the PS 603_T2 (Figure 3B3), 20 genes were shared between NAA and Pollen. Notably, three genes were under-expressed in the NAA treatment: TRINITY_DN23722_c2_g1, TRINITY_DN7013_c2_g1, and TRINITY_DN261446_c1_g2 (Putative uncharacterized protein ART2).

**FIGURE 4:**
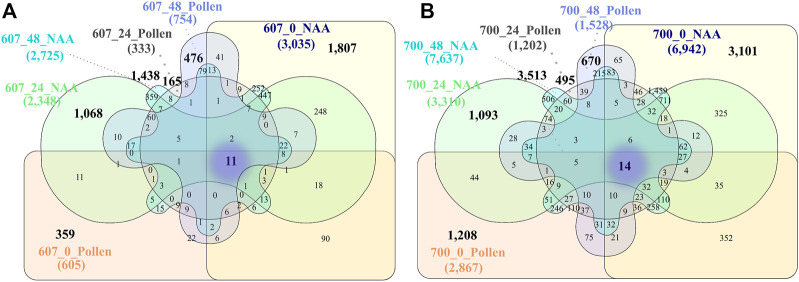
**(A)** General Venn diagrams for PS 607. **(B)** General Venn diagrams for PS 700. Unique genes for the phenology stage are highlighted in bold, and shared genes among all phenology stages and times are highlighted in the purple fog. Conventions: 603_48_Pollen = Phenology stage_time post treatment_treatment.

The Venn diagrams for PS 607 represented 11 shared genes among T0, T1, and T2 between NAA and Pollen treatment (Figure 4A). From those, two genes presented relevant changes at the expression level between NAA and Pollen: TRINITY_DN141433_c0_g1 and TRINITY_DN2888_c0_g2 (possible protein Lateral Root Primordium 1-like). The PS 700 shared 14 DEGs among T0, T1, and T2, and NAA and Pollen treatment (Figure 4B). From those, two genes presented a relevant change in expression level between NAA and Pollen: TRINITY_DN795_c0_g2 (possible Alpha-galactosidase-1) and TRINITY_DN1989_c2_g2.

Several TF families were identified between NAA and Pollen treatment. The expression of the most relevant TF families such as WRKY, Ethylene-responsive transcription factor (ERFs), Auxin response factor (ARFs), MYB, bHLH, and Heat stress transcription factors (HS) was variable and dependent on the stage and time post-treatment (Table 1). In PS 603, all TF families mentioned above were over-expressed. But, in PS 607 and PS 700, they changed their expression levels, even in the lapse of time of 24 h. The ERF family was under-expressed in PS 607 and PS 700 under NAA treatment. WRKY was found present in all PS and times under NAA treatment. The PS 700_T0 point showed the same TFs between Pollen and NAA. However, the expression was inverse among them.

**TABLE 1:** Dynamics of the main TF expressed under Pollen and NAA treatment in oil palm interspecific O × G hybrids.

Stage	Time	Pollen	NAA
603	T0	**↑**WRKY, **↑**ERF, **↓**ARF, **↑**MYB, **↑**bHLH	**↑**WRKY, **↑**ERF
	T1	**↑**bHLH	**↑**WRKY, **↑**MYB, **↑**bHLH
	T2	**↑**WRKY, **↑**ERF, **↑**ARF, **↑**HS	**↑**WRKY, **↑**ERF, **↑**ARF
607	T0	**↓**ERF, **↓**MYB, **↓**HS	**↑**WRK, **↓**ERF, **↑**ARF, **↓**MYB
	T1	**↓**WRKY, **↓**MYB, **↑**ARF	**↓**WRKY, **↓**ERF, **↑**ARF, **↓**MYB, **↓**HS
	T2	**↓**ERF, **↑**ARF	**↑**WRKY, **↓**ERF, **↓**ARF, **↑**MYB, **↑**bHLH
700	T0	**↑**WRKY, **↑**ERF, **↓**ARF, **↑**bHLH, **↓**HS	**↓**WRKY, **↓**ERF, **↑**ARF, **↓**bHLH, **↑**HS
	T1	**↓**WRKY, **↑**ARF, **↑**MYB, **↓**bHLH, **↑**HS	**↑**WRKY, **↓**ERF, **↑**ARF, **↑**MYB, **↓**bHLH, **↓**HS
	T2	**↑**WRKY, **↓**ARF, **↓**MYB, **↓**HS	**↑**WRKY, **↓**ERF, **↓**ARF, **↑**MYB, **↓**bHLH, **↑**HS

Conventions: **↑**, over-expressed TF family; **↓**, under-expressed TF family; ERF, ethylene-responsive transcription factor; ARF, auxin response factor; HS, heat stress transcription factor; bHLH, basic helix-loop-helix.

Concerning GO terms enrichment (Supplementary Table S3), REVIGO categorized the PS 603 under NAA treatment into biological process terms such as organic and aromatic compound biosynthesis, response to stimulus, macromolecules biosynthesis process, and organic cyclic compound biosynthetic process. The general enrichment for PS 603 under Pollen treatment involved the cellular amide metabolic process, intracellular signal transduction, and transcription. In PS 607, the most frequent functional annotations for NAA treatment were response to stimulus, signaling, and the catabolic process cellular response to stress. The most frequent terms for Pollen treatment in PS 607 were ncRNA Processing, cellular protein metabolic process regulation, and aminoglycan metabolic process. The most frequent terms for the PS 700 under NAA were cellular biosynthetic process, cellular nitrogen compound biosynthetic process, and macromolecule biosynthetic process. This stage under Pollen treatment was categorized with terms such as catabolic process, DNA recombination, and cell division.

### Validation of DEGs by RT-qPCR

The expression of 13 DEGs was validated by RT-qPCR analysis. RT-qPCR data highly correlated with the RNA-Seq values predicted for each gene (Supplementary Figure S2). Four genes were over-expressed under NAA treatment: Probable WRKY transcription factor 33 (TRINITY_DN143542_c1_g1), 1-aminocyclopropane-1-carboxylate oxidase (TRINITY_DN11220_c0_g2), NAC domain-containing protein 68 (TRINITY_DN4308_c0_g2), Acetyl-coenzyme A carboxylase carboxyl transferase subunit alpha, chloroplastic (TRINITY_DN38599_c0_g1). Meanwhile, the genes Flowering-promoting factor 1-like protein 1 (TRINITY_DN23510_c0_g1) and TRINITY_DN63896_c2_g1 were found over-expressed under Pollen treatment. TRINITY_DN63896_c2_g1 has an unknown function and presented the highest HUB score in the Pollen co-expression network but was not significant in NAA treatment. The genes Indole-3-acetic acid-induced ARG7-like (XM_010942329.2), Auxin-responsive protein SAUR71-like (XM_010943300.2), Flavin-containing monooxygenase 1 (XM_019853925.1), Auxin-induced protein 22D (TRINITY_DN12260_c0_g1), Gibberellin-regulated protein 14 (TRINITY_DN123905_c0_g1) and Auxin-responsive protein IAA9 (TRINITY_DN3523_c0_g1) were found under-expressed in NAA treatment. While in Pollen treatment, the transcription factor MYC2 (TRINITY_DN62356_c0_g1) was under-expressed.

## Discussion

We studied the transcriptome of three flower phenology stages of the interspecific O×G hybrid Coari x La Me, involved in the parthenocarpic fruit formation induced by NAA. The PS 603, or pre-anthesis III is the phenology stage before anthesis. In this stage, we expected to find initial changes in the molecular mechanism of parthenocarpic fruit development. The PS 603 co-expression network for the NAA application showed a non-modular form (round shape) compared with the PS 603 co-expression network of the Pollen application (Figures 2A, D). A possible biological interpretation is the simultaneous activation of several pathways caused by the exogenous application of NAA in inflorescence. After pollen application, we did not observe critical gene expression changes across the times (T0, T1, and T2). However, we found over-expressed genes involved in plant growth, adaptation, flowering, and fruit ripening, such as class I Heat Shock proteins, Alcohol dehydrogenase 1, and Tubulin alpha-3 chain (Jin et al., 2016; ul Haq et al., 2019). In both Pollen and NAA treatments, the transcriptions factors Ethylene-responsive and Probable WRKY transcription factor 33 were over-expressed across times T0 to T2 in PS 603, suggesting a possible mediation of natural processes (Table 1).

An important fact to understand the parthenocarpic phenomena is to analyze the proteins involved in the reception of the stimuli. In this sense, we identified receptor-like protein kinases RLKs over-expressed in the NAA treatment compared to the Pollen treatment in PS 603_T0. We hypothesize that probable receptor-like protein kinase At1g11050 responds to the exogenous application of NAA and activates components of the calcium-conducting subunit (Calcium uniporter protein 5). A signal mediated by secondary messengers, such as calcium, ROS, or H_2_O_2_, induces response gene expression. Similar results were found in the eggplant transcriptome analysis, where two calcium-binding proteins, PBP1, were upregulated in the natural parthenocarpic line PP05 (Chen et al., 2017). Following this signal, we compared the NAA and Pollen treatments in the PS 603_T0 and found the gene 4-coumarate—CoA ligase 2 (4CL2) over-expressed. This gene has been reported in producing CoA thioesters and phenolic compounds in *Arabidopsis* (Ehlting et al., 1999). In *E. guineensis*, synergic over-expression of 4CL2 and MAD-box TF activate the expression of programmed cell death genes and induce the formation of seedless phenotype (Htwe et al., 2022). The exogenous application of NAA might be perceived as a chemical stress and immediately triggers a response against this stress. Thus, the identified stimuli receptors and early candidate response genes might play an essential role in parthenocarpic fruit development in the O×G hybrid.

At 24 h post-NAA treatment (PS 603_T1), genes related to stress (Stress-related protein) and plant-pathogen interactions (Subtilisin-like protease SBT1.5) were over-expressed. These findings reinforce the chemical stress hypothesis mentioned above. However, four non-annotated genes were dramatically under-expressed in this stage compared with the Pollen application: TRINITY_DN261449_c1_g1, TRINITY_DN47630_c1_g1, TRINITY_DN261946_c0_g1, and TRINITY_DN65993_c0_g1. The gene co-expression network grouped these genes by expression profile in common modules; additionally, NCBI BLAST^®^ (https://blast.ncbi.nlm.nih.gov/Blast.cgi) and Interpro (https://www.ebi.ac.uk/interpro/) alignments did not show significative results. Considering the expression level, further studies will be necessary to identify the role of these genes in the NAA-induced parthenocarpic process in the interspecific O×G hybrid. After 48 h of Pollen application (PS 603_T2), we observed over-expressed genes involved in cell wall biogenesis, such as Beta-galactosidase 2 and Alcohol dehydrogenase 1. Their presence could be related to the natural formation of reproductive structures. In contrast, under NAA treatment, the mechanism of the cell wall formation might be affected by the under-expression of genes related to the xylem cell wall thickening, Cellulose synthase A catalytic subunit 7. In this stage, the response to stimuli and organic cyclic compound metabolic process were the most common GO terms (Supplementary Table S2).

During anthesis (PS 607), processes co-occur around fertilization. Pollen tube elongation, pollen deposition on the stigmas, nuclear fusion, gametophyte development, and anther dehiscence are some of the main processes (Drews and Yadegari, 2002). Under Pollen treatment in PS 607, we identified a subset of genes that control some plant developmental processes, such as the transition from flower to seed and the female gametophyte development. The APETALA2/Ethylene responsive factor (AP2/ERF), NUCLEAR FUSION DEFECTIVE1 (NFD1) gene, and SCARECROW gene were found over-expressed in T0. Those genes are required for gametophyte development and cell specification of surrounding stem cells (Wysocka-Diller et al., 2000; Portereiko et al., 2006). At 24 h (T1) of Pollen treatment in PS 607, the gene ABC transporter D family member 1 (ABCD1) was over-expressed. This gene has been found in the peroxisomal membrane importing substrates into the peroxisome for *β*-Oxidation and enhancing fertilization (Footitt et al., 2007). Finally, at 48 h (T2), the gene MADS-box transcription factor 6 (MADS6) involved in floral patterning and gametophyte development was found over-expressed, too (Ohmori et al., 2009).

In contrast, no genes related to fertilization and gametophyte development described above were significant under NAA treatment in PS 607. Instead, we found many stress-related genes and enzymes modulating oxidative stress and senescence mechanisms. Among them, the genes serine/threonine-protein kinase (At1g54610), trans-resveratrol di-O-methyltransferase (ROMT), subtilisin-like protease SBT1.1 (SBT1.1) and peroxidases 18 (PER18), formerly reported in biotic and abiotic stress response, were found over-expressed in T0. ROMT, SBT1.1, and PER18 were induced by UV light and AlCl3 treatments in grape and pathogen infections in *Arabidopsis* (Schmidlin et al., 2008; Figueiredo et al., 2014). In T1, the genes chalcone synthase 4 (CHS4), SBT1.1, superoxide dismutase [Fe] 2 (sodM), PER18, and Protein ACCELERATED CELL DEATH 6 (ACD6) were found over-expressed as well. The CHS4 genes have been reported in flavonoid/isoflavonoid biosynthesis in plant defense (Dao et al., 2011), while sodM and PXG4 catalyze radical anion scavenging and remove H_2_O_2_, respectively. The gene ACD6 plays a vital role in plant response to abiotic stresses and nitrogen remobilization (Jasinski et al., 2021). Remarkably, in T2, genes related to defense against pathogens such as G-protein coupled receptor (Protein CANDIDATE G-PROTEIN COUPLED RECEPTOR 2 CAND2), receptor-like protein EIX2 and nitrate transporters (Protein NRT1/PTR FAMILY 6.3 NPF6.3) were found over-expressed. Interestingly, in PS 607, some genes related to vascular development and functioning and abaxial cell fate in flower meristems were common and highly over-expressed between NAA and Pollen treatment. The gene Dof zinc finger protein 4 (DOF4) acts at the crosstalk of various developmental pathways in vascular plants, while the YABBY 3 (YAB3) gene acts redundantly to specify abaxial identity in *Arabidopsis* (Siegfried et al., 1999; Le Hir and Bellini, 2013). In this sense, the NAA treatment favors the transition from flower to seed, activating stress-related genes and nitrogen remobilization pathways. These processes could disturb the molecular mechanism involved in the development of reproductive organs but do not affect the mechanism involved in the functional and structural fruit process.

The post-anthesis, or PS 700, presented the most DEGs among all stages. The female flower is fertilized, the stigma turns black, and the fruit development begins. At this point, the control of cell division and expansion are critical factors for early fruit development. At 24- or 48 h post-pollination begins the seed filling and the exponential growth of the fruit until the next 45 days (Hormaza et al., 2012). Under Pollen treatment, we observed a subset of genes related to the biosynthesis of phospholipids, long-chain fatty acid catabolic pathway, senescence, and expression of several TF in T0. The genes Acyl-CoA-binding domain-containing protein 3 (ACBP3), Peroxisomal (S)-2-hydroxy-acid oxidase GLO4 (GLO4) Probable linoleate 9S-lipoxygenase 4 (LOX1.4) were found highly over-expressed. On the other hand, the TF probable WRKY transcription factor 72 (WRKY72), ethylene-responsive transcription factor 71 (ERF071), NAC domain-containing protein 58 (NAC58), and transcription factor bHLH162 were found over-expressed as well. Among them, the TF WRKY72 has been reported in the abscisic acid signal and auxin transport pathway in *Arabidopsis* inflorescence (Yu et al., 2010), the TF ERF071 has been related to sweet cherry fruit development (Alkio et al., 2014) and with the cell expansion during olive fruit development (Camarero et al., 2023). However, NAC58 and bHLH162 have not been reported in fruit development.

At 24 h post Pollen treatment, we observed genes involved in calcium signaling in the cytosol, such as Calcium-dependent protein kinase 5 (CDPKs). Curiously, all the TF expressed in T0 were not differentially expressed in T1. Instead, other TF such as NAC domain-containing protein 4 (NAC4), transcription factor MYB78, and heat stress transcription factor B-1 (HSFB1) were over-expressed. At 48 h, we found over-expressed the genes GATA transcription factor 16 (GATA16) and thaumatin-like protein (TLP1), both are associated with developmental processes, tissue differentiation, and induced systemic resistance (Reyes et al., 2004; Singh et al., 2013). The pollination process in the oil palm O × G in PS 700 triggers signaling processes, carbohydrate catabolic processes, cell differentiation, and cell division. These processes are involved in flower maturation and fruit development and could be modulated by TF WRKY72, ERF071, NAC58, and bHLH162 in the first 24 h post-pollination.

Regarding NAA treatment in PS 700_T0, a structural molecular activity associated with flower transition, senescence, and sulfate transport was observed. The gene family Squamosa promoter-binding-like protein (SPL), auxin response factor 6 (ARF6), and Cyclic dof factor 2 (CDF2) were identified in this stage. These genes have been associated with flower and fruit development, cereal grain yield improvement, and senescence (Nagpal et al., 2005; Chen et al., 2010). Additionally, the gene Sulfate transporter 2.1, (SULTR2.1), which plays a vital role in the uptake of sulfate into *Arabidopsis* seeds, was found over-expressed (Awazuhara et al., 2001). The expression of auxin response factor 7 (ARF7), auxin response factor 12 (ARF12), probable WRKY transcription factor 33 (WRKY33), transcription factor bHLH162, EPIDERMAL PATTERNING FACTOR-like protein 2 (EPFL), and protein ASPARTIC PROTEASE IN GUARD CELL 1 (ASPG1) genes In T1, suggest an essential role in the plant epidermal cell growth process, dormancy, germination of the seed and viability.

Finally, in PS NAA_700_T2, the genes GIGANTEA, FAR1-RELATED SEQUENCE 6 (FRF), Gibberellin 2-beta-dioxygenase 8 (GA2ox8), and MYB78 were the most relevant over-expressed. GIGANTEA and FRF have been involved in flowering time regulation, light signaling, sucrose signaling, and starch accumulation (Mishra and Panigrahi, 2015; Ma and Li, 2018). While GA2ox8 has been related to the degradation of bioactive gibberellin in seedless citrus fruit (Zhang et al., 2017). The exogenous application of NAA in this stage accelerates the transition from flower to fruit. The activation of genes that control flowering and light could trigger senescence, while the activation of the starch metabolism could induce cell growth and seed filling. The reduction of GAs could be co-related with the previously described process and promote the development of the oil palm hybrid seedless genotype.

### Essential genes involved in parthenocarpic fruit development

The PS 607 is the phenological stage of flowering. Here, several hormones regulate fruit development and determine the path of senescence or fruit development depending on the signaling process mediated by auxins or ethylene (An et al., 2020). Consistent with the knowledge about signal pathways involved in parthenocarpy phenomena, we emphasize the role of ARF, SAUR, IAA, and GA genes. The expression of ARFs plays an essential role in the plant’s growth and control of different developmental processes (Pandolfini et al., 2009). Comparative transcriptome analyses for parthenocarpic fruit development in some vegetables, such as eggplant, tomato, and cucumber, show that genes related to auxin signaling are the most affected (Chen et al., 2017; Li et al., 2017; Zhang et al., 2021).

The gene ARF8 presented differences among the treatments. Under NAA treatment, ARF8 was under-expressed in the post-anthesis (PS 700) stage for T0 and T1, but in Pollen, the treatment was not significant for DESeq. ARF8 has been reported as a critical negative regulator involved in parthenocarpic fruits in eggplant. It inhibits the formation of the AUX/IAA9 complex, blocks the fertilization process, and can activate the fruit initiation genes (Du et al., 2016). In contrast, the ARF7 was strongly over-expressed at post-anthesis (PS 700) for T1 under NAA treatment, but in Pollen, the treatment was not significant for DESeq. This is a particular finding because ARF7 mediates cross-talk between auxin and gibberellin signaling during tomato fruit set and development (De Jong et al., 2011). Okabe et al. (2019) recently observed that the expression of SlARF7 in tomatoes, encoding a negative regulator of auxin signaling, sharply decreased after anthesis. In our case, the expression of ARF7 was different, and further analysis should be followed to identify the role of this gene in NAA-induced parthenocarpy.

The SAUR is a family of auxin-responsive genes critical to plant growth, development, stress responses, and cell elongation in *Arabidopsis* (van Mourik et al., 2017; Stortenbeker and Bemer, 2019). Our results showed that SAUR genes exhibit a negative regulation of gene expression under NAA treatment at anthesis (PS 603) across the three time periods evaluated and did not show any relevant performance for the other stages/treatments. Similar results were reported in oil palm (*E. guineensis*), where SAUR71-like genes were downregulated in the auxin treatment, compared with controls (Somyong et al., 2018). In zucchini (*Cucurbita pepo* L.) parthenocarpy transcriptome, SAUR gene expression decreased under pollination and auxin treatment (Pomares-Viciana et al., 2019). In tomatoes, the SAUR genes showed an upregulated expression in parthenocarpic tomato line “R35-P” and downregulated expression in non-parthenocarpic tomato lines “R35-N” “R35-N” at −2 days after anthesis (Zhang et al., 2021). Therefore, our results confirm that this gene family is involved in generating parthenocarpic fruits in the O×G hybrid, and unknown factors inhibit the positive regulation.

According to Luo et al. (2018), the Auxin/Indole-3-Acetic Acid (Aux/IAA) gene family has been identified as short-lived nuclear proteins that control the expression levels of genes activated by the auxin response factor (ARF) family and exhibit an essential role in parthenocarpic fruit development. This study showed a more significant response of Aux/IAA genes (at least 12) under-expressed in pre-anthesis (PS 603) under NAA treatment across the three sample times. In contrast, only two Aux/IAA genes were expressed at PS 700 for T0 and T1. In the Pollen treatment, Aux/IAA genes tended to be under-expressed at post-anthesis PS 700_T0. These results show that the downregulated expression for IAA is a typical response across different plant species (Chai et al., 2019; Molesini et al., 2020). Furthermore, the over-expression of the oil palm EgDREB1 gene in tomatoes increased production of LeAux/IAA and may inhibit the transcription of auxin-associated genes and prevent fruit set due to failure in the fertilization process (Azzeme et al., 2017). In the transcriptome analysis of oil palm inflorescences treated with the synthetic auxin, 2, 4, 5-tri-chlorophenoxy propionic acid (2, 4, 5-TP), the EgARG7 gene was under-expressed in auxin-treated samples (Somyong et al., 2018), which corresponded with our results. Also, for the zucchini (*C. pepo* L.) parthenocarpy transcriptome, 10 Aux/IAAs were expressed differently, whereas IAA26 and IAA18 were found to downregulated in parthenocarpic and non-parthenocarpic cultivars (Pomares-Viciana et al., 2019).

Gibberellins (GAs) are critical phytohormones for germination, shoot elongation, tuber formation, flowering, and fruit set process (Serrani et al., 2007). Exogenous applied GAs can induce parthenocarpic fruits in species such as tomatoes, eggplant, apples, and others (Pandolfini et al., 2009; Galimba et al., 2019). In addition, the expression of GA genes has been studied in artificially induced parthenocarpy to understand their role in signaling pathways. In this study, the transcriptional profile of five isoforms of GA genes was under-expressed in the NAA treatment at post-anthesis (PS 700) across T0, T1, and T2. In contrast, only one isoform was downregulated at anthesis (PS 607) for T2, and two isoforms were downregulated for PS 700 for T0 and T1.

GA genes responded late to NAA and Pollen treatments in the O×G hybrid. Galimba et al. (2019) observed similar results in' Honeycrisp' apples. RNA expression profiles (clustered expression patterns of DEGs) were highly similar in GA3-induced and hand-pollinated fruits. In zucchini, a subset of genes associated with GAs biosynthesis modulated during pollen treatment showed downregulation patterns (Pomares-Viciana et al., 2019). On the other hand, applications with auxins also displayed some shared GAs genes with similar under-regulation patterns. In tomatoes, Okabe et al. (2019) showed a crosstalk between GA and auxin and suggested that stamen development negatively regulates fruit set by repressing the GA biosynthesis. These results reinforce the relevant role of GA genes for fruit set and parthenocarpy, independently of the species or auxin treatment. Additionally, in our study, the high quantity of GA genes expressed at post-anthesis (PS 700) under NAA application compared with Pollen could explain the effectivity of auxin for parthenocarpy fruit development when the anthesis stage has been finished.

## Conclusion

**FIGURE 5:**
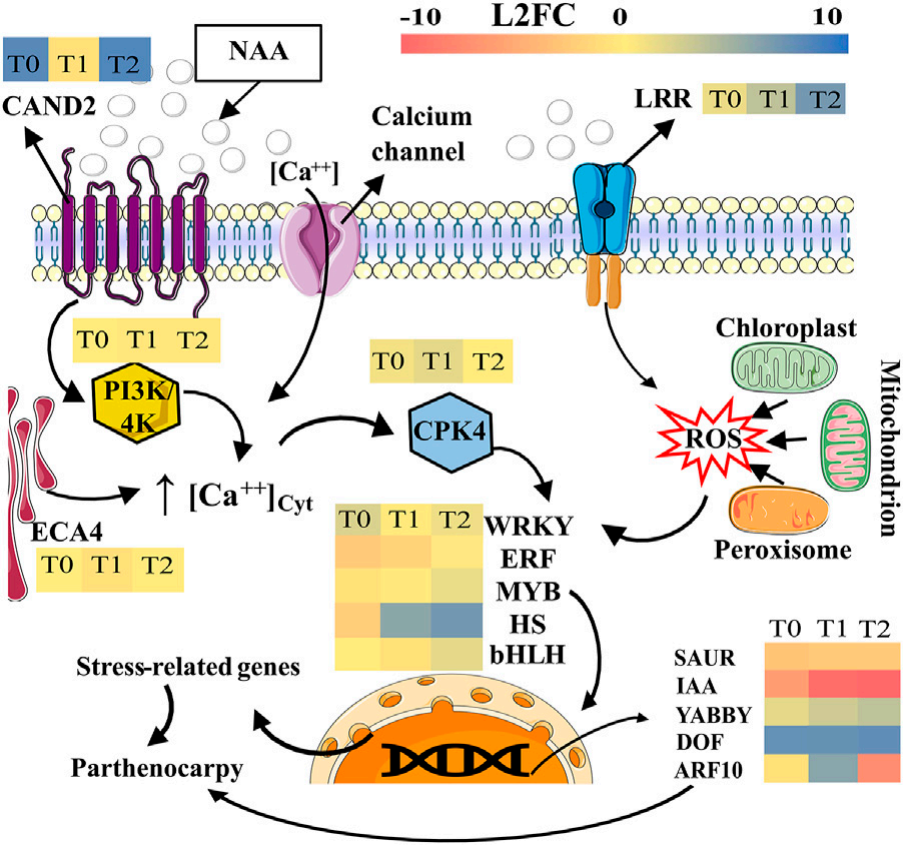
Possible model to understand de parthenocarpy fruit development induced by NAA in oil palm interspecific O × G hybrids at the anthesis (PS-607). Gene expression levels are indicated for treatments T0 (5 min post-treatment), T1 (24 h post-treatment) and T2 (48 h post-treatment). The color scale corresponds to the L2FC (upper right). Conventions: CAND2, Protein candidate G-protein coupled receptor 2; CPK4, Calcium-dependent protein kinase 4; PI3K/4K, 1-phosphatidylinositol-3-phosphate 5-kinase; ECA4, Calcium-transporting ATPase 4, endoplasmic reticulum-type, and LRR, Probable LRR receptor-like serine/threonine-protein kinase At5g10290. The role of genes such as WRYK, ERF, MYB, HS, bHLH, SAUR, IAA, YABBY, DOF, and ARF10 are detailed in the discussion section. The figure was partly generated using Servier Medical Art, provided by Servier, licensed under a Creative Commons Attribution 3.0 unported license (https://creativecommons.org/licenses/by/3.0/; https://bioicons.com).

The gene expression comparison between Pollen and NAA-treated inflorescences at PS 603 to induce parthenocarpic fruits revealed candidate genes involved in the early response of parthenocarpic induced by exogenous auxin. In PS 607, the inhibition of genes involved in karyogamy, the regulation of TF, and the under-expression of auxin response genes in NAA treatment, might accentuate the parthenocarpic phenomena induced by NAA (Figure 5).

Our findings agreed with the transcriptomic results published about parthenocarpy phenomena and the predicted signal pathways (Htwe et al., 2022; Sharif et al., 2022). Critical genes such as ARF, ERF, SAUR, IAA, and GA exhibited transcriptional profiles similar to those reported previously, which implies that the oil palm O × G hybrids have similar genetic mechanisms of response to exogenously applied auxins to induce parthenocarpy (Somyong et al., 2018; Htwe et al., 2022).

The validation of candidate genes and HUBs found in this study by RT-qPCR confirms that the transcriptome is reliable and supports the findings of Daza et al. (2020) and Romero et al. (2021) for induction of parthenocarpic fruits in interspecific O×G hybrids. It is especially worthwhile to pinpoint the role of the HUB genes in fruit set and development, which could be a target for genome editing techniques to facilitate the production of parthenocarpic fruits free of plant growth regulator applications.

## Data Availability

The original contributions presented in the study are publicly available. This data can be found here: https://www.ncbi.nlm.nih.gov/. Accession number: PRJNA901998.

## Supplementary material

SUPPLEMENTARY FIGURE S1 General Venn diagrams for PS 603. Unique genes for the phenology stage are highlighted in bold, and shared genes among all phenology stages and times are highlighted in the purple fog. Conventions: 603_48_Pollen = Phenology stage_time post treatment_treatment.

SUPPLEMENTARY FIGURE S2 Validation of 13 genes by RT-qPCR. Association between RNA-Seq and RT-qPCR.

SUPPLEMENTARY TABLE S1 Primer sequences used for RT-qPCR validation.

SUPPLEMENTARY TABLE S2 Summary metrics of co-expression networks.

SUPPLEMENTARY TABLE S3 Top 20 GO terms by phenology stage under NAA and Pollen treatment.
