# Supplementary figures and images for: Parthenocarpy-related genes induced by naphthalene acetic acid in oil palm interspecific O × G [Elaeis oleifera (Kunth) Cortés × Elaeis guineensis Jacq.] hybrids
Source: Front Genet. 2023 Mar 20;14:1099489. doi: 10.3389/fgene.2023.1099489 (PMC10067579; doi:10.3389/fgene.2023.1099489)

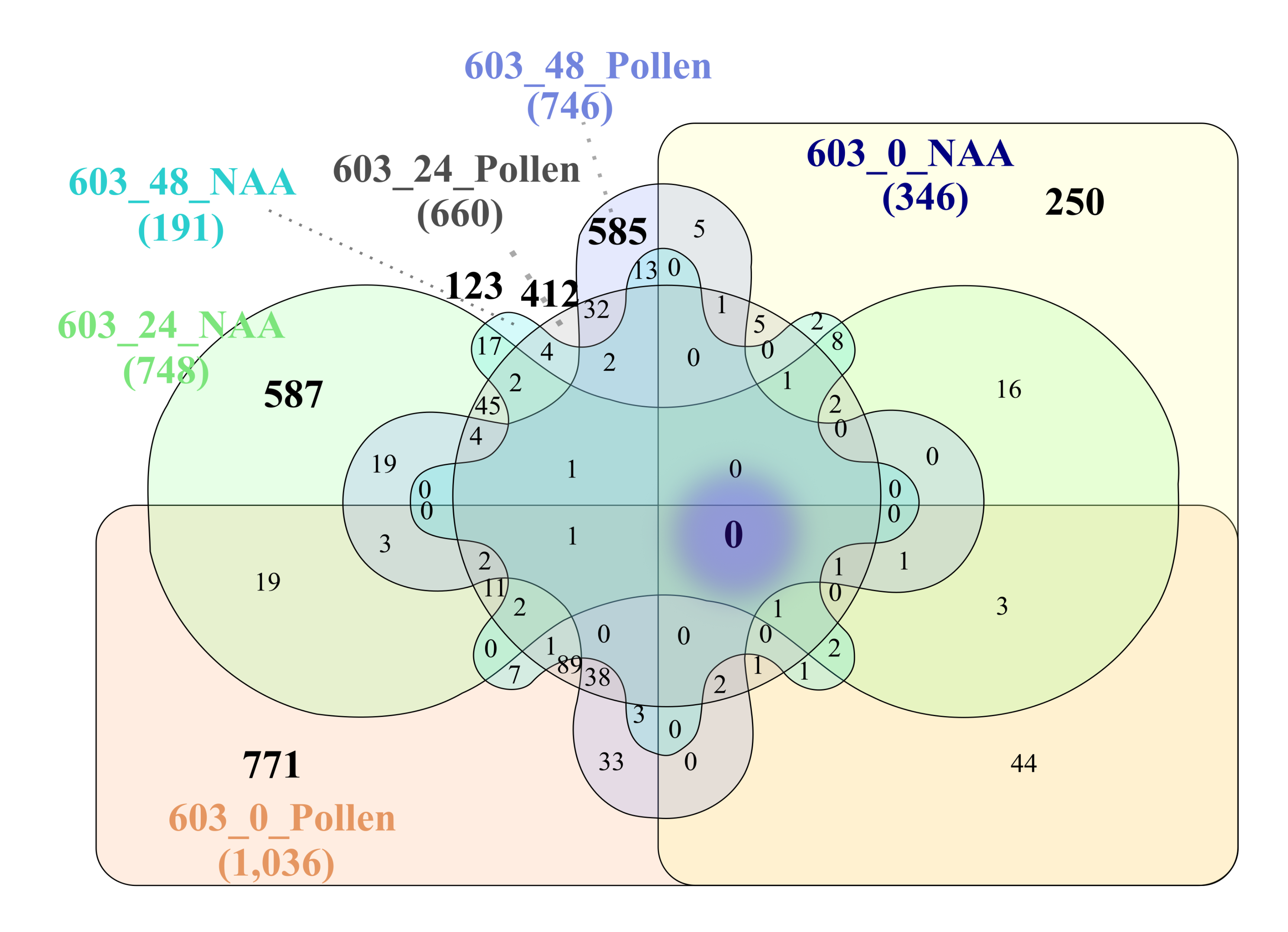

Supplement: Supplementary file 2 [file Image1.TIFF]

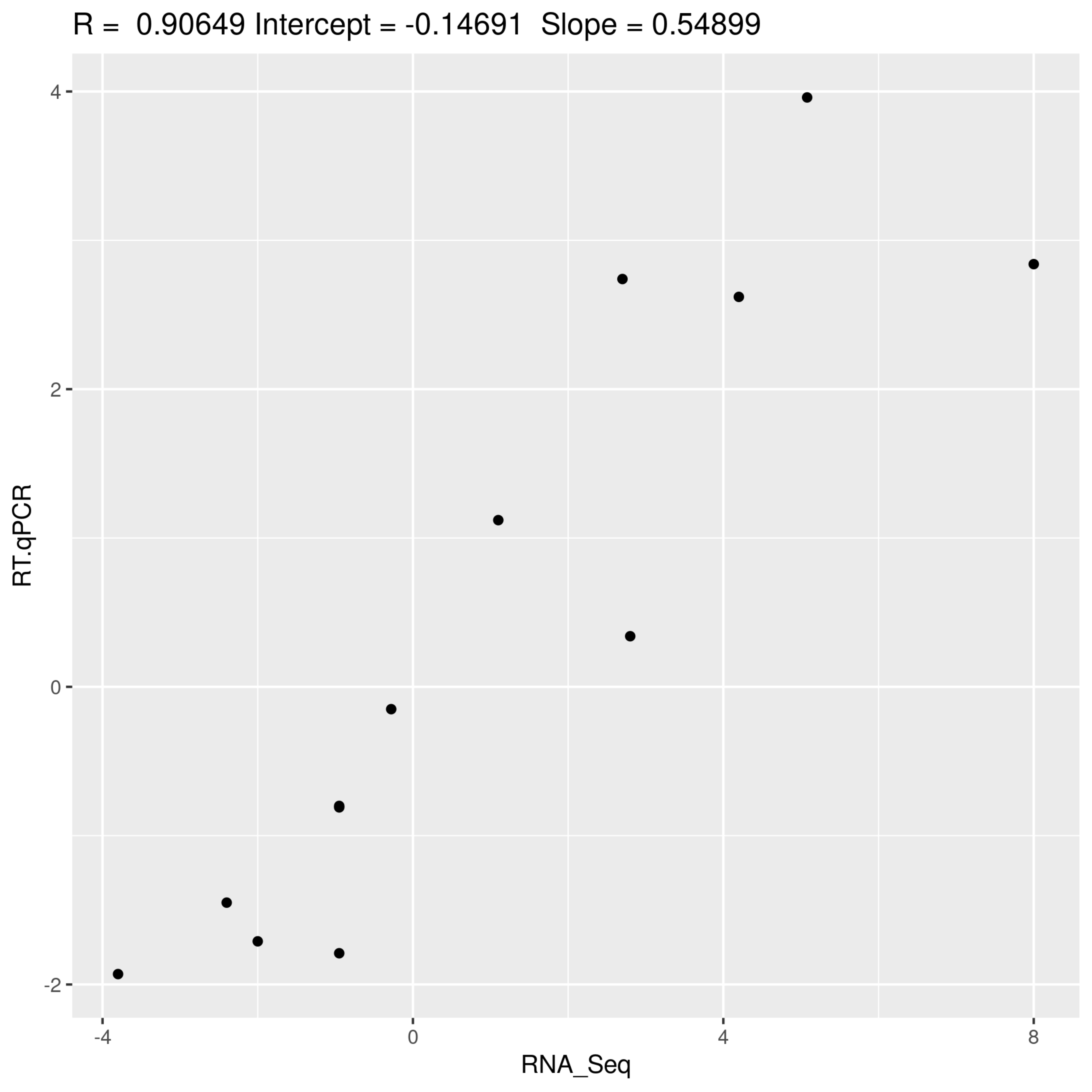

Supplement: Supplementary file 5 [file Image2.TIFF]
